# Endophytic *Trichoderma*: Potential and Prospects for Plant Health Management

**DOI:** 10.3390/pathogens13070548

**Published:** 2024-06-28

**Authors:** Dimitrios Natsiopoulos, Eleni Topalidou, Spyridon Mantzoukas, Panagiotis A. Eliopoulos

**Affiliations:** 1 Plant Health Management Lab, Department of Agrotechnology, University of Thessaly, 41500 Larissa, Greece; 2 Hellenic Agricultural Organization DIMITRA, Forest Research Institute, 57006 Thessaloniki, Greece; etopal@fri.gr; 3 Department of Agriculture, University of Ioannina, 47100 Arta, Greece; sdmantzoukas1979@gmail.com

**Keywords:** endophytic fungi, *Trichoderma*, disease management, pest control

## Abstract

The fungus *Trichoderma* is widely regarded as the most common fungal biocontrol agent for plant health management. More than 25 *Trichoderma* species have been extensively studied and have demonstrated significant potential in inhibiting not only phytopathogen growth but also insect pest infestations. In addition to their use as biopesticides, there is increasing evidence that several *Trichoderma* species can function as fungal endophytes by colonizing the tissues of specific plants. This colonization enhances a plant’s growth and improves its tolerance to abiotic and biotic stresses. In recent decades, there has been a proliferation of literature on the role of *Trichoderma* endophytes in crop protection. Although the mechanisms underlying plant–fungal endophyte interactions are not yet fully understood, several studies have suggested their potential application in agriculture, particularly in the mitigation of plant pests and diseases. This review focuses on the diversity of *Trichoderma* endophytic strains and their potential use in controlling specific diseases and pests of crop plants. *Trichoderma* endophytes are considered a potential solution to reduce production costs and environmental impact by decreasing reliance on agrochemicals.

## 1. Fungal Endophytes

The term “endophyte” comes from the Greek, endo- (=within) and -phyto (=plant) and it was first discovered by Anton de Bary in 1866, as an organism found within the tissues of living plants. Non-parasitic fungi are very common endophytes and generally develop a symbiotic relationship with the plants they colonize. Symbiosis is a term used to describe the association between two different organisms, where at least one organism benefits from the other. When a microorganism enters inside the host tissue, the host’s reaction depends on the nature of the invader. If the endophytic fungi (EF) are non-parasitic—symbiotic—then it will be a balanced interaction between the two organisms and an asymptomatic response of the host, whereas the parasitic fungi creates symptoms [1]. Specifically, non-pathogenic EF are those which inhabit plants internally, without causing any visible disease symptoms to their hosts. They change the plant metabolism, thereby altering the tolerance and protecting plants against pests and diseases [2].

Many endophytic microorganisms enter plant tissues and interact with the host in different ways, either negatively (parasitism), or neutrally for one or both organisms. Mutualism is the type of symbiosis where both organisms benefit. Around 300,000 different plant species host one or more EF [3]. Symbiotic fungi have a significant impact on a plant’s health and its tolerance to biotic and abiotic stress factors. Mutualistic endophytes may contribute to drought tolerance, growth promotion, and pest and disease resistance [4].

Unlike mycorrhizal fungi that colonize and develop only in plant roots, endophytes can be found within various plant compartments, such as roots, stems, wood, leaves, flowers, and fruits [5,6]. There are two main categories of EF, the Clavicipitaceous and the non-Clavicipitaceous endophytes. The Clavicipitaceae family belongs to the phylum Ascomycota and colonizes shoots and roots of plants of the Poaceae family. These fungi develop between plant tissues with a mutualistic interaction for both organisms. On the other hand, the non-Clavicipitaceous are fungi that belong to other orders beside Ascomycota, are more diverse, and are associated with a wide variety of plants. They can be isolated from non-vascular plants, conifers, and angiosperms [7].

EF diversity depends on geographic distribution and the host type, and they differentiate their functioning according to the plant they colonize and the different environments they are exposed to. For example, the root-colonizers require moisture, darkness, and organic matter. In contrast, foliar EF can tolerate UV radiation and dry conditions [8]. It is estimated that there are more than one million different EF species producing a large number of different compounds that have beneficial effects on their hosts, in terms of resistance to biotic and abiotic stress [9,10]. Marine algae are also a source of several EF. Twenty-six different fungal species belonging to Ascomycota and Basidiomycota have been isolated from ten algae species and demonstrated antimicrobial activity [11]. 

A plethora of beneficial EF have been isolated from a huge variety of plants in many recent studies [10]. For example, ninety-seven EF isolates have been identified and isolated from stems and leaves of two-year oil seed rape cultivation, belonging to forty different species, many of them having antifungal activity against certain pathogens [12]. Apart from that, more than two hundred EF from twenty-one different genera and thirty-eight different species were isolated from the Liquorice *Glycyrrhiza glabra* L. (Fabales: Fabaceae), demonstrating fungicidal effects on *Fusarium* and *Phoma*. Moreover, they all produce plant hormones, in different amounts, with a positive impact on plant development [13]. 

Apart from stimulating plant growth and disease management, the action of EF can lead to control of insect pests [14,15,16] or even nematodes [17]. Additionally, many EF species have been exploited to manage abiotic stress in plants under conditions of drought, salinity, etc. [18].

The fungus *Trichoderma* has been known and studied for many years for its antagonistic activity against plant pathogenic fungi [19], while commercial biopesticides based on various Trichoderma strains are also available on the market. However, when it was found that, in addition to being a soil saprophyte, it could also penetrate and colonize plant tissues, studies were initiated on its exploitation as an endophyte [20]. Because of its importance as a plant protection agent, the diversity of *Trichoderma* endophytic strains and their potential as biocontrol agents against specific plant diseases and pests are described in detail below.

## 2. Endophytic *Trichoderma*

The genus *Trichoderma* (Hypocreales) is cosmopolitan and includes ubiquitous species which are found in diverse environments. They commonly colonize a wide range of niches, including soil, above-ground plant tissues (microflora), dead wood, sediment organic matter, and various other substances [21]. *Trichoderma* spp. include well-known soil saprophytic fungi which exhibit several antifungal properties. They act in the rhizosphere against numerous soil pathogens and nematodes, through various modes of action (mycoparasitism, nutrient competition, etc.). However, many species that have been isolated from plant tissues develop symbiotic associations and effects on plant tolerance to biotic and abiotic stress factors. Endophytic *Trichoderma* have been focused for many years on root colonization; however, many studies indicate that they may also occur in aboveground plant tissues [20,22]. 

### 2.1. Endophytic Trichoderma Diversity

Although most Trichoderma species have been isolated from different soil types and dead plant tissue, endophytic Trichoderma species have been isolated from living plant tissues from a wide variety of plants as well (Table 1). In recent decades, *Trichoderma* spp. have been increasingly isolated as endophytes from both herbaceous and woody plants, and they tend to show habitat specialization. Therefore, species isolated as endophytes are not typically found in soils, and vice versa [21].

The use of new molecular tools which are provided by the “omics” sciences can contribute significantly to the understanding of plant–Trichoderma interaction mechanisms and the influences of the environment on the observed/recorded phenotypic response of the plant to the colonization by the endophytic Trichoderma spp. According to Hu et al. [23], diversity and taxonomic structure of Trichoderma spp. are largely influenced by the ecosystem, climate, and biogeographic patterns. Although various surveys have been conducted to study the diversity of the Trichoderma genus, novel species are continuously reported from new regions, ecological niches, and hosts. High diversity is recorded especially in perennial crop plants and wild trees which live in their original wild-to-semi-wild ecosystems [24].

**Table 1:** Endophytic *Trichoderma* species isolated from plant tissues.

*Trichoderma* Species	Host Plant	Plant Part ^a^	Reference
*T. acreanum*	*Hevea* spp.	*	[21]
*T. afroharzianum*	*Pandanus* sp.	R	[25]
	*Triticum aestivum*	R	[26,27]
*T. ararianum*	*Hevea* spp.	*	[21]
*T. asperelloides*	*Adinandra* sp.	R	[25]
	*Bambusa* sp.		
	*Cymbopogon citratus*		
	*Dimocarpus fumatus*		
	*Durio* sp.		
	*Eucalyptus pellita*		
	*Homalomena* sp.		
	*Ixora coccinea*		
	*Koompassia excelsa*		
	*Mimosa pudica*		
	*Paspalum* sp.		
	*Piper nigrum*		
	*Psidium guajava*		
	*Saccharum officinarum*		
	*Shorea* sp.		
	*Turnera subulata*		
	*Veitchia merrillii*		
*T. asperellum*	*Alstonia* sp.	R	[25]
	*Andira inermis*		
	*Bambusa* sp.		
	*Cyrtostachys renda*		
	*Elaeis guineensis*		
	*Eusideroxylon zwageri*		
	*Helianthus* sp.		
	*Hymenocallis littoralis*		
	*Melastoma malabathricum*		
	*Turnera subulata*		
	*Saccharum* spp.	R, L	[28]
	*Theobroma cacao*	F, W	[29]
*T. atroviride*	*Cupressus sempervirens*	T	[30]
	*Cupressus arizonica*	L	
	*Juniperus excelsa*	L	
	*Hevea* spp.	*	[21]
	*Coffea* sp.	L	[5]
	*Terminalia catappa*	B	[31]
	*Ananas comosus var. bracteatus*	S	[32]
	*Astronium fraxinifolium*	Sp	
*T. botryosum*	*Coffea arabica*	L, F, S	[5]
	*Coffea canephora*	S	
*T. brasiliensis*	*Hevea* spp.	*	[21]
*T. breve*	*Coffea canephora*	S	[5]
	*Coffea arabica*	F	
*T. brevicompactum*	*Allium sativum*	*	[33]
*T. caeruloviride*	*Coffea arabica*	F	[5]
*T. caribbaeum*	*Theobroma gileri*	Tr	[34]
*T. erinaceum*	*Hevea* spp.	*	[21]
*T. guizhouense*	*Vatica micrantha*	R	[25]
	*Coffea* sp.	S	[5]
	*Triticum aestivum*	R	[27]
*T. hamatum*	*Coffea arabica*	S, F	[5]
	*Theobroma gileri*	F	[35]
*T. harzianum*	*Acacia crassicarpa*	R	[25]
	*Acacia mangium*		
	*Acranthera* sp.		
	*Adinandra* sp.		
	*Alstonia* sp.		
	*Bambusa* sp.		
	*Campnosperma* sp.		
	*Casuarina* sp.		
	*Dryobalanops beccarii*		
	*Durio griffithii*		
	*Elateriospermum tapos*		
	*Eucalyptus pellita*		
	*Eurycoma longifolia*		
	*Excoecaria agallocha*		
	*Ficus* sp.		
	*Ilex* sp.		
	*Musa* sp.		
	*Neolamarckia cadamba*		
	*Palaquium* sp.		
	*Shorea* sp.		
	*Sindora* sp.		
	*Tristaniopsis whiteana*		
	*Xanthophyllum* sp.		
	*Triticum aestivum*	R	[26]
	*Ananas comosus var. bracteatus*	S	[32]
	*Astronium fraxinifolium*	Sp	
	*Bowdichia virgilioides*	Sp	
	*Caesalpinia pyramidalis*	Sp	
	*Glycine max*	S	[36]
*T. heveae*	*Hevea* spp.	*	[21]
*T. konigiopsis*	*Combretum laxum*	L	[37,38]
	*Myrcia tomentosa*		
	*Hevea* spp.	*	[21]
	*Coffea canephora*	S, L	[5]
	*Ananas comosus var. bracteatus*	S	[32]
	*Astronium fraxinifolium*	Sp	
	*Bowdichia virgilioides*	Sp	
	*Caesalpinia pyramidalis*	Sp	
*T. koningii*	*Cypressus sempervirens*	T	[30]
*T. lentiforme*	*Hevea* spp.	*	[21]
*T. lentissimum*	*Coffea arabica*	S	[5]
*T. longibrachiatum*	*Combretum glaucocarpum*	L	[37,38]
	*Posidonia oceanica*	R	[39]
	*Saccharum* spp.	R	[28]
	*Ananas comosus var. bracteatus*	S	[32]
	*Astronium fraxinifolium*	Sp	[32]
	*Bowdichia virgilioides*	Sp	[32]
	*Caesalpinia pyramidalis*	Sp	[32]
*T. orientale*	*Cenostigma macrophyllum*	L	[37,38]
*T. ovalisporum*	*Hevea* spp.	*	[21]
	*Banisteriopsis caapi*	S	[40]
*T. parareesei*	*Coffea arabica*	S	[5]
*T. pseudopyramidale*	*Coffea arabica*	S, L	[5]
*T. reesei*	*Alstonia* sp.	R	[25]
	*Amorphophallus* sp.		
*T. simmonsii*	*Triticum aestivum*	R	[26]
*T. sinuosum*	*Ananas comosus var. bracteatus*	S	[32]
	*Astronium fraxinifolium*	Sp	[32]
*T. sparsum*	*Hevea* spp.	*	[21]
*T. spirale*	*Hevea* spp.	*	[21]
	*Coffea canephora*	S	[5]
*T. strigosum*	*Tristaniopsis* sp.	R	[25]
*T. theobromicola*	*Coffea canephora*	S	[5]
	*Theobroma cacao*	Tr	[41]
	*Cola praecuta*	Tr	[42]
*T. virens*	*Acacia mangium*	R	[25]
	*Baccaurea motleyana*		
	*Bambusa* sp.		
	*Calamus* sp.		
	*Casuarina equisetifolia*		
	*Cleistanthus* sp.		
	*Cratoxylum* sp.		
	*Dipterocarpus* sp.		
	*Elaeis guineensis*		
	*Eusideroxylon zwageri*		
	*Ficus variegata*		
	*Garcinia mangostana*		
	*Ixora coccinea*		
	*Koompassia excelsa*		
	*Lansium parasiticum*		
	*Macaranga gigantea*		
	*Melastoma* sp.		
	*Metroxylon sagu*		
	*Nauclea* sp.		
	*Neolamarckia cadamba*		
	*Pandanus amaryllifolius*		
	*Hevea* spp.	*	[21]
	*Coffea brevipes*	S	[5]
	*Caesalpinia pyramidalis*	Sp	[32]
*Τ. viride*	*Spilanthes paniculata*	R	[43]
*Trichoderma* sp.	*Bauhinia cheilantha*	L	[37,38]
	*Cordia toqueve*		
	*Diptychandra aurantiaca*		
	*Mimosa tenuiflora*		
	*Pityrocarpa moniliformis*		
	*Calophyllum* sp.	R	[25]
	*Duabanga moluccana*		
	*Durio* sp.		
	*Koompassia malaccensis*		
	*Musa campestris*		
	*Shorea* sp.		

^a^ R: Root, S: Stem, L: Leaf, W: Wood, T: Twig, Sp: Sapwood, F: Fruit, B: Bark, Tr: Trunk, *: not mentioned.

As illustrated in the table above, endophytic *Trichoderma* are mainly root colonizers, but sometimes are isolated from the leaves or stem. Much rarer are the cases of their establishment in wood (twig, trunk), whereas in the case of fruits, they have been found only in coffee and cocoa beans (Table 1).

The distribution of host species significantly affects the biodiversity of the *Trichoderma* isolates. Several strains of *T. atroviride* and *T. koningii*, isolated from Cuppressaceae plants, have shown strong antifungal potential against *Diplodia seriata* and *Phaeobotryon cupressi* and antibacterial effect against *Pseudomonas syringae*, *Erwinia amylovora*, and *Bacillus* spp. [30]. *Trichoderma* isolates have also been collected from the root surface of fifty-eight genera of thirty-five families from Malaysian habitats, with *T. asperellum*, *T. asperelloides*, *T. harzianum*, and *T. virens* the most common species, suggesting many of them as potential biocontrol agents [25].

EF isolated from leaves and branches have been found in mangrove (*Rhizophora mangle* and *Avicennia schaueriana* trees) habitats. *Trichoderma* was one of the most abundant genera, isolated from 8.72% of samples from leaves and branches, and showed great biocontrol potential against *Fusarium* rot [44]. 

Nineteen strains from two different habitats in Brazil, one from a savanna (Cerrado) and the other from an area with rich biodiversity (Caatinga), were isolated from leaves of forest trees. The *Trichoderma* species were identified as *T. orientale*, *T. longibrachiatum*, *T. koningiopsis*, and six isolates assumed to be new species. 

Endophytic Trichoderma appear to show great diversity in host specificity. Several species (*T. harzianum*, *T. virens*, *T. atroviride*, and others) have been isolated from a plethora of plants of different genera and families, while others have been found only in one species or plant family. It is quite common for endophytes to show variable degrees of host specificity, ranging from highly specialized relationships with only one plant species, to simpler associations with many different hosts [45]. Apparently, the host specificity demonstrated by endophytic microorganisms can be very complex, and although Trichoderma–plant relationships can be formed naturally, they also be manipulated and result in a new association. More studies are needed to fully understand these complex relationships and exploit them to develop biological crop protection formulations.

### 2.2. Pest Control

Although the main target of *Trichoderma* are soil pathogenic fungi, certain isolates can also act against insect pests via several mechanisms, direct or indirect, such as plant defense induction, the production of enzymes that affect moths’ midgut structure, the production of secondary metabolites with pesticidal properties, and the release of compounds from plants that attract natural enemies or act as pest antifeedants [46,47]. Although *Τrichoderma* has the capacity for direct insecticidal action through the production of insecticidal compounds, the endophytic strains appear to act by indirect mechanisms [46]. The most common is the activation of plant defensive pathways, not locally but systematically. These responses are regulated by various hormones like jasmonic and salicylic acid [14,48,49]. Some *Trichoderma* strains, after colonizing plant tissues, initiate the production of VOCs that may attract natural enemies, mainly parasitoid wasps [16,50]. Another indirect insecticidal mechanism of endophytic *Trichoderma* is the parasitism of symbiotic fungi necessary for the survival of certain insects [51]. 

The application of endophytic *Τrichoderma* to cultivated plants often requires artificial inoculation of the plant. This can be achieved in various ways, which often influences the final outcome. Seed coating, soil drenching, foliar spraying, and seedling dipping have been mainly tried for *Trichoderma* colonization [52,53]. However, it should be mentioned that the effect of the inoculation method on the efficacy of *Trichoderma* as a biocontrol agent has not been studied, but only the effect on the promotion of plant growth.

The few cases where endophytic *Trichoderma* have acted as entomopathogens are presented in Table 2.

Specifically, the endophytic *T. hamatum* isolated from kale roots was evaluated against *S. littoralis* larvae and compared with a commercial *B. bassiana* strain. Larvae were treated with conidial solution through spraying (10^10^ conidia/mL) and orally (10^8^ conidia/mL) through the diet. In the first case, mortality was 43–50% of both fungi with no significant differences between them. In the oral application, the mortality was significantly higher (85%) for *T. hamatum* compared with *B. bassiana* (77%) [56]. 

Control of the maize pest *S. frugiperda* by endophytic *T. atroviride* strain IMI 206040 was achieved by inoculating maize seedlings with the combination of a wasp parasitoid *Campoletis sonorensis* in terms of a multitrophic interaction system. The results revealed that there was a positive interaction between the endophytic *Trichoderma* strain and the parasitoid. Wasp parasitism was significantly higher (50%) when seedlings were inoculated with *T. atroviride* due to the release of the secondary metabolite 6-pentyl-2H-pyran-2-one (6-PP) of the fungus, suggesting that it acts as a parasitoid attractant, enhancing its activity. Additionally, these volatiles acted as antifeedants, resulting in significantly lower feeding of the pest (55%) compared to the non-inoculated seedlings [48].

The tomato leaf miner *Tuta absoluta* is a difficult pest to control in tomato cultivation, even with conventional pesticides. However, several alternative to chemicals methods can be considered effective. Endophytic *T. asperellum* M2RT4 isolated from maize plant tissues was applied in tomato seeds by soaking in a spore suspension of 10^8^ conidia/mL and then planted in pots. Fungal colonization was assessed by plating plant tissues in PDA and showed that *T. asperellum* colonized about 80% of plant tissues. Oviposition and egg hatching of *T. absoluta* was significantly lower in plants inoculated with *T. asperellum* M2RT4 compared with *B. bassiana*, *T. atroviride* F2S21, *Hypocrea lixii* F3ST1, and the control (untreated plants). Pupation was also affected negatively by *T. atroviride* compared to the control [15]. Bioassays proved that *T. absoluta* avoids tomato plants inoculated with EF due to the emission of methyl selicylate. Moreover, *T. asperellum* M2RT4 alters tomato physiology and produces (Z)-jasmone and activates the production of additional methyl selicylate, which is considered a repellent semio-chemical [14].

Apart from moth larvae, endophytic *Trichoderma* have shown promising results against sucking pests such as aphids, thrips, and whiteflies. Significant decrease in *Thrips tabaci* population was observed on onion plants inoculated by EF strains of *T. asperellum* (isolated from sorghum), *T. atroviride*, and *T. harzianum* (isolated from onion) as compared to the untreated control [44]. Similarly, when *T. harzianum* was applied to tomato plants (soil drenching and foliar spraying) followed by infestation by *Bemisia tabaci*, increase in the developmental period, lower settling, and oviposition preference, and fewer eggs were recorded [57]. The common aphid pest *Macrosiphum euphorbiae* has been effectively controlled on tomato plants by inoculation with an endophytic strain of *T. atroviride* [49]. 

### 2.3. Disease Management

The genus *Trichoderma* is widely recognized as parasite of other fungi and includes well-studied species as biocontrol agents against several soil-borne diseases. *Trichoderma* is not only associated with phytopathogenic fungi in many ways, such as competition, hyperparasitism, and antibiosis, but also interacts with many soil microorganisms like bacteria and nematodes [58]. 

The biocontrol mechanisms of *Trichoderma* are based on the activation of multiple biochemical pathways; they compete for space and nutrients within plant tissues, and many species have the ability to control pathogen development by producing secondary metabolites which have limited toxicity to plants and exhibit antimicrobial properties (Table 3). The best-studied are antibiosis, mycoparasitism, and induced resistance, while others remain unknown [20]. In mycoparasitism, *Trichoderma* is in close proximity to the target and can penetrate the other fungi, forming appresoria [59,60], although this remains unclear for endophytic strains [20]. Another mechanism, termed antibiosis, involves the production of secondary metabolites, such as gliotoxin, that are toxic to fungal phytopathogens but not to plants [61]. The most common mechanism is induced resistance in which *Trichoderma*–plant associations contribute to plant disease control. It is well documented that when *Trichoderma* colonizes plant tissue (mainly roots), it triggers responses in the plant that prevent further colonization [60]. In this way, protection against phytopathogens is also provided in other parts of the plant (leaves, branches) where *Trichoderma* may not be present. Apart from that, production of enzymes and secondary metabolites (such as alkaloids, terpenoids, phenols, propanoids, and quinines) are industrially processed and used to induce systemic resistance in plants and promote plant growth [21].

In another assay, *Trichoderma longibrachiatum*, *T. harzianum*, and *T. pleuroti* were isolated from *Leucas aspera* (Lamiaceae), *Pterocarpus santalinus* (Fabaceae), and *Juniperus lutchuensis* (Cupressaceae). All strains were evaluated for the mechanism of antibiosis against *Sclerotinia sclerotiorum*, *Sclerotium rolfsii*, *Fusarium oxysporum*, and *Macrophomina phaseolina*. Firstly, all four isolates inhibited pathogens 90% by mycoparasitism, except *M. phaseolina*. In single cultures, volatile organic compounds (VOCs—alcohols, aldehydes, esters and ketones) were detected from *T. longibrachiatum* strain and the pathogens *F. oxysporum* and *M. phaseolina*. However, in dual cultures, *Trichoderma* produced VOCs that were not produced in single cultures, with 45–50% effectiveness against *S. sclerotiorum*, *S. rolfsii*, and *F. oxysporum* [69]. On the other hand, *T. longibrachiatum*, isolated from rice, inhibited *M. phaseolina* by 58% in a dual culture trial, due to the production of metabolites. The concentration of compounds such as 1-6-anhydro-’a-D-glucopyranose and 5-heptyl dihydro-2(3H)-furanone was increased when the two fungi interacted in dual cultures. The metabolites indicated that antibiosis is the reason for inhibited growth of *M. phaseolina* [71].

*Phytophthora cinnamomi* was efficiently controlled by endophytic *Trichoderma* isolated from avocado and cinnamon tree roots. Specifically, three species and nine strains were identified: *T. asperellum* (4 strains), *T. harzianum* (3 strains), and *T. koningiopsis* (2 strains). All strains were evaluated against avocado root rot *P. cinnamomi* under in situ and in vitro conditions. In vitro dual culture tests with *T. asperellum* T-AS2 and *T. harzianum* TH-3 demonstrated the highest *P. cinnamomi* inhibition rate: 78.3% and 73.33%, respectively, while better colonization rates were recorded for *T. koningiopsis* T-K11 (67.83%) and *T. harzianum* T-H3 (60%). During in situ experiments, avocado seedlings were inoculated with *P. cinnamomic*. All *Trichoderma* strains contributed to a reduction in disease of more than 50%. However, *T. asperellum* T-AS7 achieved 80% reduction, *T. asperellum* T-AS2 75%, and *T. koningiopsis* T-K11 77%, compared to the control [62].

*Phytophthora palmivora* leaf fall disease is an important disease of rubber trees, that has been managed by endophytic *Trichoderma* isolated from healthy rubber leaves. Five species were isolated: *T. asperellum*, *T. harzianum*, *T. hamatum*, *T. longibrachiatum*, and *T. virens*, and fifty-nine different strains were identified. The spore solution was sprayed on rubber leaves and afterwards, they were inoculated with *P. palmivora*. Among all isolates, *T. harzianum* KUFA0436 and KUFA0437 were most effective in reducing leaf symptoms by 43% and 48%, respectively, under glasshouse conditions [63].

A new species, identified as *T. phayaoense*, was isolated from Siam weed leaves and has been very effective against muskmelon gummy stem blight caused by *Stagonosporopsis cucurbitacearum* and wilt caused by *Fusarium equiseti*. In dual culture trials, the inhibition rates were 81.6% and 90.8%, respectively, and the overgrowth on the pathogen colony showed that besides antagonism, parasitism was also a mode of action of this biological agent. In pot experiments, *T. phayaoense* have reduced wilt disease severity 80% and 60% on gummy stem blight compared with the control plants [73].

Endophytic *Trichoderma* were also evaluated against banana wilt caused by *F. oxysporum* f.sp. *cubense* isolated from healthy roots of banana trees. The species were identified as *T. harzianum* (3 strains), *T. asperrellum*, *T. gamsii*, and *T. koningiopsis*. During in vitro dual culture experiments, all the strains inhibited *Fusarium* growth by 50–60%. In planta experiments, *T. asperellum* and *T. harzianum* have demonstrated wilting symptoms down to 8.3%, whereas the control symptoms were 78%, 5 weeks after treatment. Given that phenolic compounds from the roots of both species were increased, it is suggested that endophytic *Trichoderma* reduced wilt symptoms through induced resistance [64].

In some cases, endophytic *Trichoderma* were more effective against sugarcane *Fusarium sacchari* wilt compared to soil-borne isolates. Although pathogen growth was reduced by 26.3% in dual cultures with *Trichoderma* spp. SER 10, the inhibition was enhanced through the food poisoning method, where metabolites inhibited *Fusarium* growth by 44.22% [75]. Six different species and seven strains were isolated from rice leaves, with *T. longibrachiatum* being the most effective against many phytopathogens such as *M. phaseolina*, *Magnaporthe grisea*, *Pythium* sp., *R. solani*, *F. oxysporum*, and *Colletotrichum falcatum*, with inhibition rates ranging from 60 to 82% [72].

An endophytic *Trichoderma* was isolated from the medicinal plant *Leucas aspera* and identified as *T. confertum*, which can colonize the roots and root tips the Thale cress *Arabidopsis thaliana*. Tests in dual cultures with *Alternaria brassicicola* showed 75% coverage of the pathogen colony by the *Trichoderma* hyphae with an hyperparasitic mode of action. Moreover, it has been proven to be an endophytic biological agent that acts as a protectant when inoculated prior to or after *Alternaria* infection of cress seedlings. In both cases, *Alternaria* symptoms were significantly reduced [68].

*Armillaria* root rot is a soil pathogen difficult to be managed. The use of protective endophytic biological agents could be an efficient method for strawberry and privet plants [76]. Forty different strains of *Trichoderma* were isolated from a variety of symptomatic plants. All isolates were screened on strawberry plants to evaluate their potential as biocontrol agents. *Trichoderma* inoculation was carried out by dipping plant roots in conidial suspension of 10^5^ conidia/ml for two minutes, and two months later, soil was inoculated with *Armillaria*. The colonization efficacy six weeks after root inoculation was 92–98%. Seven isolates proved to have protective potential against *Armilaria* root rot, and two *T. atrobrunneum* strains significantly reduced disease severity for strawberry and privet plants [66].

Endophytic *Trichoderma* strains from wheat roots were evaluated for the control of *Fusarium graminearum* and for plant growth. Among 54 isolates, the best performance was recorded by the *T. harzianum* T136 strain, which caused >85% inhibition rate. All other strains did not exceed 58% [27]. Another fusariosis, *Fusarium guttiforme*, a major threat to pineapple cultivation worldwide, has been effectively managed in the field by *Trichoderma* endophytes isolated from various plants in Brazil. From 109 tested strains, one isolate of *Τ. atroviride* and two of *T. koningiopsis* were very successful, given that they decreased disease severity by 68–84% [32].

Strains isolated from leaves of Brazilian forest trees (six new species and *T. orientale*, *T. longibrachiatum*, *T. koningiopsis*) showed inhibitory activity of 50–70%, 30–78%, 49–78%, and 2–69% against *Colletotrichum truncatum*, *Lasiodiplodia theobromae*, *Macrophomina phaseolina*, and *Sclerotium delphinii*, respectively, in dual culture tests. Moreover, fourteen *Trichoderma* isolates produced secondary metabolites with an antibiosis mode of action against *C. truncatum* [38]. Endophytic *T. longibrachiatum*, isolated from roots of seagrass *Posidonia oceanic*, controlled *Pythium ultimum* efficiently in melon seedlings under saline soil conditions at a concentration of 2 g/L NaCl [39].

Grapevine plants have also been a source of several fungal endophytes with antifungal potential against trunk diseases. *Trichoderma* isolates have shown great potential as biocontrol agents due to their ability to grow rapidly, sporulate, and produce secondary metabolites [77,78,79,80,81,82,83,84]. Ten *Trichoderma* strains of six different species were isolated from healthy plants from a vineyard with a high population of trunk-diseased symptomatic plants. All strains were tested for their biocontrol potential in dual cultures against *Aphanomyces cochlioides*, *Pythium acantophoron*, *Botryosphaeria dothidea*, *Diaporthe eresand*, the grapevine trunk diseases, *Diplodia seriata*, *Eutypa lata*, and *Neofusicoccum parvum*. In vitro antagonism tests showed that the biocontrol index of *T. afroharzianum* strain TR04 was 90–100% for all pathogens. *Trichoderma simmonsii* TR05 had a similar biocontrol index, but for *B. dothidea*, it was significantly lower, at 25% [78]. A mixture of a spore suspension of the endophytic isolates *T. simmonsii*, *T. orientale*, and *T. gamsii* and a second mixture of *T. afroharzianum* and *T. simmonsii* were used in order to inoculate new vine rootstocks with the soaking method. The total loss of inoculated plants after four years was 30% lower than for the non-inoculated. Moreover, *Trichoderma* strains were successfully isolated from the transplanted vines five to fifteen months after treatment [85]. Additionally, endophytic *Trichoderma* (Altair 607QR6), isolated from grapevine trunk, demonstrated 78% growth inhibition and 100% overgrowth and mycoparasitism against *D. seriata* and *N. parvum* after 21 days. The inhibition rate was also evaluated by a lignified tissue method, which recorded 100% growth inhibition rate of the two pathogens when grapevine shoots were pre-inoculated with *Trichoderma*, whereas the fungicide Tebuconazole had no impact on either pathogen [84]. 

## 3. Conclusions

Climate change and the need for reduction of the use of chemical pesticides has facilitated the development of alternative management methods to control plant pests and diseases. The use of beneficial EF represents a promising and innovative method for developing natural alternatives to the use of chemical pesticides and for attaining environmental sustainability. Endophytic *Trichoderma* spp. and the products derived from those species (metabolites, enzymes, etc.) are considered among the best alternatives towards the development of sustainable methods of controlling fungal diseases because such compounds can modulate the plant microbiome and prevent invasion of pathogenic microbes [86]. Until recently, species of *Trichoderma* were assumed to act against pathogens primarily through mycoparasitism, antibiosis, or antagonism (for space and resources). However, their ability to produce a mixture of secondary metabolites has revealed their attributes to confer selective advantages to their hosts (e.g., plant resistance to abiotic stresses, increased plant growth, disease resistance, etc.). In this review, endophytic isolates of the genus *Trichoderma* were presented for their proven effects against various plant pathogens (*Phytophthora* spp., *Armillaria* spp., *Diplodia seriata*, *Botryosphaeria* spp., etc.) and insect pests (moths, thrips, whiteflies, etc.) revealed their potential role in promoting plant health directly or indirectly. 

Although the host plant recognizes the fungal invasion by both endophytes and pathogens, the response to the invaders is quite different [87]. EF and host interactions involve complex mechanisms such as metabolite production, gene expression, and hormonal signaling molecules that activate plant resistance [88]. Endophytic *Trichoderma* employ different and diverse mechanisms in plant growth promotion and protection. This review reported the ability of endophytic *Trichoderma* to act as bioinoculants which boost plant tolerance to biotic and abiotic stress factors. Nevertheless, the pathways that endophytic *Trichoderma* enter the plant endosphere and the mechanisms involved in the interaction with their hosts demand clarification by researchers. However, the biotechnological importance of endophytic *Trichoderma* was demonstrated in this review, through the report of the production of secondary metabolites, which stimulate antibiosis against plant pathogens and cause alterations in the morphology and physiology of the host. 

Metabolomic analysis successfully differentiated *Trichoderma* strains and identified secondary metabolites which exhibited significant biocontrol potential against crop pathogenic fungi [38]. It is well known that the mutualistic associations of EF with their hosts is controlled by the genes of both organisms and modulated by the environment in which they live [38]. Microbe-associated molecular patterns (MAMPs) are involved in the local or systemic responses of the plants to all invasive micro-organisms (mutualist or pathogenic) [89], which result in the triggering of enhanced/induced plant defenses [90]. Even though various MAMPs have been identified, the systemic resistance triggered by EF is yet not well understood, because systemic changes in gene expression are either mild or not easily detectable [90]. Endophytic *Trichoderma* spp., occurring naturally in the wild with other groups of fungi, exhibit a number of characteristics (antagonism for nutrients, production of secondary metabolites, mycoparasitism, etc.) which indicate their use as potential tools for the control of important plant diseases. However, the main obstacle in their development as biocontrol tools relates to the lack of understanding of their interaction with the variable climatic and environmental conditions of real crops (and/or ecosystems) and their ability to multiply efficiently under varied conditions and persist even under unfavorable conditions.

The great advantage of endophytic *Trichoderma* spp. is their ability to easily colonize their host plants and persist within them. Provided that *Trichoderma* spp. occur endophytically before the infection by certain pathogens, the chances of protective control should be increased due to the mutualistic relationship developed with the host plant. In addition, many endophytic *Trichoderma* spp. have been reported for their effects against insects. However, endophytic occurrence of *Trichoderma* spp. in terms of plant responses to the dual attack by insects and pathogens has never been studied, and offers an interesting area for the investigation of multi-level interactions among different organisms (plant-fungi-insects).To date, the failure of *Trichoderma* spp. to effectively control plant pathogenic fungi in nature is linked either to their reduced virulence against certain plant pathogens, or to their specificity to a particular pathogen. As demonstrated in this review, much work has been conducted towards the selection of the most virulent *Trichoderma* spp., whereas there is limited knowledge and understanding on *Trichoderma* specificity—if any—to other organisms (insects and fungi) and/or the mechanisms involved in plant responses due to their colonization by endophytic *Trichoderma* spp. 

So far, *Trichoderma* spp. are used mainly as successful biocontrol agents in soil ecosystems, because soil provides a more sustainable and uniform environment for their rapid growth and development, and their ability to utilize different and diverse substrates, etc. [91]. As demonstrated in this review, different direct and indirect modes of action have been attributed to various endophytic *Trichoderma* spp. (competition for nutrients, mycoparasistism, activation of local or systemin induced plant resistance, improvement of plant growth, antibiosis, metabolite production, etc.). Despite the progress made during the past few decades, the main obstacles that limit progress are related to biological, environmental, technological, and commercial constraints. Innovative approaches and strategies are needed to consider enhancement of the resilience of the host plants through cross protection and induced resistance. However, the majority of endophytic *Trichoderma* spp. that have demonstrated such effects cannot be prepared yet in suitable formulations for widespread applications which would provide effective management of diseases and increased yield. Huge economic resources are needed for the study and deployment of efficient formulations that will ensure protection of the selected *Trichoderma* sp. from environmental stress, while acting in a robust way as chemicals do in varied conditions. 

It should be also noted that endophytic *Trichoderma* strains have additional advantages as biocontrol agents. They are adapted to the host, the agricultural environment, and certain practices (fungicide sprayings, etc.), as well as to the weather (heat, cold) and soil conditions (drought, salinity, etc). Besides that, they are not considered harmful for the soil microbiome [78]. Perhaps we need to alter the mentality of the users/producers, so as to adopt integrated management in their crops by minimizing the disturbance of natural balances and facilitate the enhancement of plant resilience. Endophytic *Trichoderma* spp. are a promising alternative for pest and disease reduction, provided that an in-depth understanding and selection could be developed for applications in different agricultural sectors under integrated disease management programs.

This review provided an update of the unique features of endophytic *Trichoderma* spp., the mechanisms that have been decoded up to date for their interactions with their host plants, and their potential role in providing protection against plant pathogens and insects of several crops.

## Figures and Tables

**Table 2:** Control of insect pests by endophytic strains of *Trichoderma*.

*Trichoderma* Species	Host Plant	Target Pest	Treatment Effect	Reference
*T. asperellum*	*Zea mays*	*Tuta absoluta*	Colonized tomato plants recorded significantly lower numbers of eggs, mines, and pupae compared to the control.	[15]
	*Sorghum bicolor*	*Thrips tabaci*	Colonized onion plants had significantly fewer thrips and feeding punctures as compared to the control.	[54]
	*Sorghum bicolor*	*Liriomyza huidobrensis*	The leafminer recorded significantly lower mean survival time (>50% reduction) and population (>70% reduction) in colonized Vicia faba plants compared to the control.	[55]
*T. atroviride*	*	*Spodoptera frugiperda*	*T. atroviride* inoculation resulted in 25% decrease of the larvae feeding on maize and consumption of significantly less leaf area. Wasp parasitism was significantly increased.	[48]
	*	*Spodoptera littoralis*	Inoculated tomato plants demonstrated negative effects on moth larval survival and development.	[49]
	*	*Macrosiphum euphorbiae*	Survival rate was significantly decreased on treated tomato plants compared to untreated ones.	
	*Allium cepa*	*Thrips tabaci*	Colonized onion plants had significantly fewer thrips and feeding punctures as compared to the control.	[54]
*T. hamatum*	*Brassica oleracea var. acephala*	*Spodoptera littoralis*	Topical treatment reached 50% mortality, while oral application was more effective (>80% larval mortality)	[56]
*T. harzianum*	*	*Bemisia tabaci*	Whitefly mortality, oviposition preference and developmental period were negatively affected by leaf and root treatment with *T. harzianum* on tomato plants. Differences were significant among treated and control plants.	[57]
	*Allium cepa*	*Thrips tabaci*	Colonized onion plants had significantly fewer thrips and feeding punctures as compared to the control.	[54]

* not mentioned.

**Table 3:** Management of phytopathogenic fungi by endophytic strains of *Trichoderma* (results from in vitro dual cultures trials and field/greenhouse tests).

*Trichoderma* Species	Host Plant	Target Pathogen	Treatment Effect	Reference
*T. asperellum*	*Persea americana*	*Phytophthora cinnamomi*	The inhibition rate of the phytopathogen by four strains in dual cultures ranged from 51 to 78%. The inoculation of avocado seedlings resulted in significant reduction (75–93%) of dead plants.	[62]
	*Hevea brasiliensis*	*Phytophthora palmivora*	In dual cultures, three strains caused inhibition of the pathogen by 55–73%. Moderate reduction of disease severity (<30%) was recorded, in greenhouse tests (spraying leaves).	[63]
	*Musa* sp.	*Fusarium oxysporum* f.sp. *cubense*	Inhibition in dual cultures reached 50%. After 5 weeks, the disease intensity was quite low (<17%)	[64]
	*Μ* *alus domestica*	*Alternaria alternata*, *Aspergillus flavus*, *Fusarium* spp., *Myrothecium verrucaria*, *Pythium aphanidermatum*, *Phytophthora cactorum*, *Phoma asparagi Penicillium brasilianum*, *Rhizoctonia solani*	The strain 6S-2 caused noteworthy inhibition rate on the growth of all phytopathogens (30–75%)	[65]
	*Saccharum* spp.	*Colletotrichum* *falcatum*	In dual culture tests, the inhibition rate was from 32.3% to 60.1%, depending on the isolate.	[28]
	*Theobroma cacao*	*Ceratobasidium theobromae*	On cacao seedlings inoculated with various isolates, disease symptoms incidence was 0–56%; on untreated seedlings it reached 88.9%.	[29]
*T. afroharzianum*	*Triticum aestivum*	*Fusarium graminearum*	In dual culture tests, inhibition percentage was 40.5%.	[26]
	*Triticum aestivum*	*Fusarium graminearum*	In dual culture tests, inhibition percentage was 58%.	[27]
*T. atrobrunneum*	*Fragaria × ananassa*	*Armillaria mellea*	Inoculated privet plants recorded significantly lower disease symptoms compared to the *Armillaria*-only control plants.	[66]
	*Quercus* sp.	*Armillaria mellea*	Strawberry plants inoculated with the strain T17/11 did not show any symptoms after their infection with *Armillaria* root rot.	[66]
	*Viburnum bodnantense*	*Armillaria mellea*	The strains T17/15 and T17/16 had a significantly lower disease severity index compared to strawberry control plants.	[66]
	*Quercus* sp.	*Armillaria mellea*	The strain T17/11 had a significantly lower disease severity index compared to *Ligustrum vulgare* plants infected with *Armillaria* root rot.	[66]
	*Viburnum bodnantense*	*Armillaria mellea*	The strain T17/15 had a significantly lower disease severity index compared to *Ligustrum vulgare* plants infected with *Armillaria* root rot.	[66]
*Τ.* *atroviride*	*Brassica napus*	*Plasmodiophora brassicae*	Symptom incidence in control rapeseed plants grown in artificially infected soil was 85–89%, and it was significantly reduced to 42–44%.	[67]
	*Terminalia catappa*	*Fusarium solani*	In dual cultures, inhibition rate was reduced by 86%. Disease severity was also reduced up to 40% in *Phaseolus vulgaris* plants.	[31]
	*Astronium fraxinifolium*	*Fusarium guttiforme*	In field tests, disease severity on pineapples decreased 81–84%.	[32]
*T. confertum*	*Leucas aspera*	*Alternaria brassicicola*	In dual culture tests, it managed to cover 75% of the pathogen colony. Inoculated cress (*Arabidopsis thaliana*) seedlings recorded significantly reduced symptoms.	[68]
*T. gamsii*	*Musa* sp.	*Fusarium oxysporum* f.sp. *cubense*	Inhibition in dual cultures reached 60%. After 5 weeks, the disease intensity was low (<25%)	[64]
*T. guizhouense*	*Triticum aestivum*	*Fusarium graminearum*	In dual culture tests, inhibition percentage was 50%.	[27]
*T. hamatum*	*Persea americana*	*Phytophthora cinnamomi*	The inhibition rate of the phytopathogen by the strain T-A12 was 51%. The inoculation of avocado seedlings resulted in significant reduction (75%) of dead plants.	[62]
	*Hevea brasiliensis*	*Phytophthora palmivora*	In dual cultures, the inhibition of the pathogen by three strains was 41–49%. They were ineffective in the greenhouse (disease reduction < 20%).	[63]
	*Sorbus aria*	*Armillaria mellea*	The strain T17/10 had a significantly lower disease severity index compared to strawberry and *Ligustrum vulgare* control plants.	[66]
*T. harzianum*	*Pterocarpus santalinus*	*Sclerotinia sclerotiorum*, *Sclerotium rolfsii*, *Fusarium oxysporum*, *Macrophomina phaseolina*	The strain MK751758 caused noteworthy inhibition rate on the growth of all phytopathogens (47–61%) except *M. phaseolina* (0%).	[69]
	*Persea americana*	*Phytophthora cinnamomi*	The inhibition rate of the phytopathogen by three strains in dual cultures ranged from 39 to 73%. The inoculation of avocado seedlings resulted in significant reduction (68–87%) of dead plants.	[62]
	*Hevea brasiliensis*	*Phytophthora palmivora*	In greenhouse tests, two strains showed significant efficacy, reducing the disease severity 43% and 48%, respectively. Under field conditions, the same strains lowered defoliation 30–33%. In dual cultures, the inhibition of the pathogen was 65–81%.	[63]
	*Musa* sp.	*Fusarium oxysporum* f.sp. *cubense*	Inhibition in dual cultures for three strains was 54–59%. After 5 weeks, the disease intensity was quite low (<17%)	[64]
	*Quercus* sp.	*Armillaria mellea*	The strain T17/08 had a significantly lower disease severity index compared to *Ligustrum vulgare* plants infected with *Armillaria* root rot.	[66]
	*Triticum aestivum*	*Fusarium graminearum*	In dual culture tests, inhibition percentage was 85.2% and 90.3% for two strains.	[27]
	*Glycine max*	*Macrophomina phaseolina*	All tested strains were successful in controlling *M. phaseolina* in dual culture tests. Colony growth was reduced, and morphological alterations were observed in the mycelia of the pathogen.	[70]
*T. koningiopsis*	*Persea americana*	*Phytophthora* *cinnamomi*	The inhibition rate of the phytopathogen by two strains in dual cultures reached 48%. The inoculation of avocado seedlings resulted in significant reduction (85%) of dead plants.	[62]
	*Musa* sp.	*Fusarium oxysporum* f.sp. *cubense*	Inhibition in dual cultures reached 55%. After 5 weeks, the disease intensity was moderate (<33%)	[64]
	*Brassica napus*	*Plasmodiophora brassicae*	Symptom incidence in control rapeseed plants grown in artificially infected soil was 85–89%, and it was significantly reduced to 47–47%.	[67]
	*Bowdichia virgilioides*	*Fusarium guttiforme*	In field tests, disease severity on pineapples decreased 68–77%.	[32]
	*Ananas comosus var. bracteatus*	*Fusarium guttiforme*	In field tests, disease severity on pineapples decreased 68–72%.	[32]
*T. longibrachiatum*	*Juniperus lutchuensis*	*Sclerotinia sclerotiorum*, *Sclerotium rolfsii*, *Fusarium oxysporum*, *Macrophomina phaseolina*	Two strains managed to inhibit the growth of *S. sclerotiorum* (40–51%), *S. rolfsii* (53–57%), and *F. oxysporum* (49–54%) in dual culture tests. However, *M. phaseolina* was not affected.	[69].
	*Oryza sativa*	*Macrophomina phaseolina*	Inhibition reached 58% in dual culture tests.	[71]
	*Hevea brasiliensis*	*Phytophthora palmivora*	In dual cultures, the inhibition of the pathogen by one strain (KUFA0442) was 39%. It proved ineffective in the greenhouse (disease reduction < 10%).	[63]
	*Oryza sativa*	*Many phytopathogenic fungi*	In dual culture trials, the strain EF5 recorded the highest growth inhibition activity against many fungal phytopathogens (23–82%).	[72]
	*Saccharum* spp.	*Colletotrichum falcatum*	In dual culture tests, the inhibition rate was from 36.5% to 66.2% depending on the isolate.	[28]
*T. olivascens*	*Rhododendron × obtusum*	*Armillaria mellea*	The strain T17/42 had a significantly lower disease severity index compared to *Ligustrum vulgare* plants infected with *Armillaria* root rot.	[66]
*T. phayaoense*	*Chromolaena odorata*	*Stagonosporopsis cucurbitacearum*	In dual culture trials, the inhibition rate was 81.6%. Disease symptoms were reduced by 60% in inoculated *Cucumis melo* seedlings	[73]
	*Chromolaena odorata*	*Fusarium equiseti*	In dual culture trials, the inhibition rate was 90.8%. Disease symptoms were reduced by 80% in inoculated *Cucumis melo* seedlings.	[73]
*T. pleuroti*	*Leucas aspera*	*Sclerotinia sclerotiorum*, *Sclerotium rolfsii*, *Fusarium oxysporum*, *Macrophomina phaseolina*	Growth of *S. sclerotiorum*, *S. rolfsii*, and *F. oxysporum* was significantly halted by all endophytes in dual culture tests (inhibition 40–65%). *M. phaseolina* was not affected.	[69]
*T. simmonsii*	*Triticum aestivum*	*Fusarium graminearum*	In dual culture tests, inhibition percentage was 58.1%.	[26]
*T. theobromicola*	*Cola praecuta*	*Phytophthora capsici*	Inoculated hot pepper seedlings that remained asymptomatic when planted in *P. capsici* infected soil were 25–60%, while control plants without symptoms were 0–10%.	[74]
*T. virens*	*Hevea brasiliensis*	*Phytophthora palmivora*	In dual cultures, two strains showed significant inhibitory effect (40–51%). They were ineffective in the greenhouse (disease reduction < 20%).	[63]
*Τ. viride*	*Spilanthes paniculata*	*Alternaria* sp., *Aspergillus* sp., *Cladosporium* sp., *Curvularia* sp., *Fusarium* sp., *Nigrospora* sp., *Penicillium* sp., *Pythium* sp. *and Trichocladium* sp.	Significant inhibitory activity was recorded in all cases except *Aspergillus* sp.	[43]
